# Evaluation of the Anti-Trypanosomal Activity of Vietnamese Essential Oils, with Emphasis on *Curcuma longa* L. and Its Components

**DOI:** 10.3390/molecules24061158

**Published:** 2019-03-23

**Authors:** Thanh Binh Le, Claire Beaufay, Duc Trong Nghiem, Tuan Anh Pham, Marie-Paule Mingeot-Leclercq, Joëlle Quetin-Leclercq

**Affiliations:** 1 GNOS Research Group, Louvain Drug Research Institute, Université catholique de Louvain, UCLouvain, 1200 Bruxelles, Belgium; claire.beaufay@uclouvain.be; 2 Department of Pharmacognosy, Hanoi University of Pharmacy, 13-15 Le Thanh Tong, Hoan Kiem, Hanoi 100000, Vietnam; tuananhpharm@hup.edu.vn; 3 Department of Botany, Hanoi University of Pharmacy, 13-15 Le Thanh Tong, Hoan Kiem, Hanoi 100000, Vietnam; ductrongeb@hup.edu.vn; 4 TFAR Research Group, Louvain Drug Research Institute, Université catholique de Louvain, UCLouvain, 1200 Bruxelles, Belgium; marie-paule.mingeot@uclouvain.be

**Keywords:** *Trypanosoma*, *Curcuma zedoaria*, *Curcuma longa*, *Litsea cubeba*, *Zingiber officinale*, α-zingiberene, β-sesquiphellandrene, *ar*-curcumene, *ar*-turmerone, curlone

## Abstract

Human African trypanosomiasis (HAT), known as sleeping sickness and caused by *Trypanosoma brucei*, is threatening low-income populations in sub-Saharan African countries with 61 million people at risk of infection. In order to discover new natural products against HAT, thirty-seven Vietnamese essential oils (EOs) were screened for their activity in vitro on *Trypanosoma brucei brucei* (*Tbb*) and cytotoxicity on mammalian cells (WI38, J774). Based on the selectivity indices (SIs), the more active and selective EOs were analyzed by gas chromatography. The anti-trypanosomal activity and cytotoxicity of some major compounds (isolated or commercial) were also determined. Our results showed for the first time the selective anti-trypanosomal effect of four EOs, extracted from three Zingiberaceae species (*Curcuma longa*, *Curcuma zedoaria*, and *Zingiber officinale*) and one Lauraceae species (*Litsea cubeba*) with IC_50_ values of 3.17 ± 0.72, 2.51 ± 1.08, 3.10 ± 0.08, and 2.67 ± 1.12 nL/mL respectively and SI > 10. Identified compounds accounted for more than 85% for each of them. Among the five major components of *Curcuma longa* EO, curlone is the most promising anti-trypanosomal candidate with an IC_50_ of 1.38 ± 0.45 µg/mL and SIs of 31.7 and 18.2 compared to WI38 and J774 respectively.

## 1. Introduction

Human African trypanosomiasis (HAT) or sleeping sickness is caused by two subspecies of the parasite *Trypanosoma brucei, T. brucei gambiense* and *rhodesiense,* while another subspecies, *T. brucei brucei* affects non-human vertebrates [1]. *T. brucei gambiense* causes the chronic form in West and Central Africa while *T. brucei rhodesiense* causes the acute form in Eastern and Southern Africa. Although many efforts were made this past decade to decrease HAT incidence, this fatal disease is still endemic in 36 African countries [2]. Remote rural areas are the most affected partly because of high poverty, higher risk of infection from the livestock reservoir (by the tsetse fly, responsible for parasite transmission, mainly living in rural areas), and lack of health care accessibility and infrastructures for current drugs administration [1]. Furthermore, it was reported recently that trypanosomes also have an extravascular localization [3,4] which makes it difficult to eliminate the disease. Moreover, available drugs for the treatment of HAT: pentamidine, suramine, melarsoprol, eflornithine, and nifurtimox, have shown not only a lot of serious side effects and limited efficacy but also the increase of drug resistance [5]. The newest product, fexinidazole, which was recommended by the European Medicines Agency in November 2018 as the first oral treatment for HAT, is however only active against infections caused by *T. brucei gambiense* [6]. So, research for alternative strategies is still needed.

Essential oils (EOs) along with other secondary metabolites extracted from plants have been used all over the world for various biological and pharmacological activities, such as antibacterial, anti-inflammatory, anti-fungal, anti-mutagenic, anti-cancer, and anti-oxidant [7]. Interestingly, EOs, due to their amphiphilic property and small molecular sizes, can cross the blood–brain barrier easily [8], which is essential to treat the neurological phase of the disease. This crossing of the blood–brain barrier constitutes a major limitation for pentamidine and suramin efficacy [9], but makes EOs become promising candidates in the development of new treatments. Indeed, a review from 2013 until April 2017 showed that 56 EOs were tested for anti-trypanosomal activity with 9 strongly and 20 moderately effective EOs [10].

However, EOs are very complex mixtures of different volatile compounds depending not only on the plant species, environmental conditions, and geographic variations, but also on other variables such as methods of harvesting, extraction, storage, and plant-related factors including parts of the plant and maturation of the plant [11]. A correct characterization of EO composition is therefore important for quality control but also for the study of the activity, toxicity, and mechanisms of action.

In the continuity of our anti-parasitic evaluation [12], thirty-seven EOs extracted from Vietnamese plants were investigated for their anti-trypanosomal activity against *Trypanosoma brucei brucei* (*Tbb*) bloodstream form. The more interesting samples were analyzed for their compositions and major components were then tested to identify active compounds.

## 2. Results

**Figure 1:**
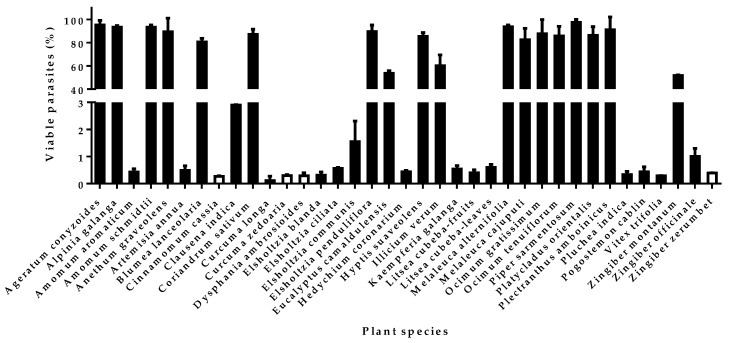
Viability percentages of *Tbb* bloodstream form treated with 25 nL/mL (in black) or 10 nL/mL (in white) of EO. Mean ± SD calculated in triplicate repeated twice and normalized according to negative control. The IC_50_ of the positive control (suramin) in those experiments was 21.53 ± 2.62 ng/mL.

Based on previous criteria (IC_50_ < 2 µg/mL: strongly effective; IC_50_ between 2 and 20 µg/mL: moderately active) [13], the thirty-seven studied EOs were firstly screened at two concentrations of 50 and 25 nL/mL (1 nL is considered to be almost 1 µg depending on the density of the EO) to identify the most active samples. Four EOs, extracted from *Cinnamomum cassia*, *Curcuma zedoaria*, *Dysphania ambrosioides*, and *Zingiber zerumbet,* were however tested at lower concentrations, 10 and 5 nL/mL, because their very volatile constituents decreased the growth of control cells in the neighboring wells or plates in the oven at higher concentrations (data not shown). This “vapor effect” was already mentioned in the study of Behar et al. [14]. The percentages of viable parasites treated at 25 (or 10) nL/mL of EO are represented in Figure 1. Nineteen EOs were determined as promising candidates for further investigations, showing less than 3% of viable parasites at the lowest tested concentration.

These nineteen EOs were then analyzed for dose-response activity on *Tbb* bloodstream form and also on mammalian WI38 and J774 cells to calculate IC_50_ values and selectivity index (SI). Three samples extracted from three Zingiberaceae species, *Curcuma longa*, *Curcuma zedoaria*, and *Zingiber officinale*, and one sample extracted from a Lauraceae species, *Litsea cubeba,* showed the most active and selective effects with IC_50_ values of 3.17, 2.51, 3.10, and 2.67 nL/mL respectively and SI > 10 compared to cytotoxicity (Table 1 in bold).

The chemical composition of these four interesting EOs was analyzed using gas chromatography (GC) with mass spectrometry (MS) and flame ionization detector (FID) in order to control their quality but also to identify some active compounds. As shown in Table 2, more than 85% of each EO composition was characterized. Monoterpenes such as citronellal (43.10%), isopulegol (11.10%), limonene (8.72%), pulegol (6.52%), linalool (5.60%), and citronellol (5.17%) were major components of *L. cubeba* EO while EOs of the three Zingiberaceae species contained mainly sesquiterpenes. Interestingly, α-zingiberene, β-bisabolene, β-sesquiphellandrene, and *ar*-curcumene were identified in both EOs extracted from *C. longa* and *Z. officinale* with relative percentages respectively of 25.38, 3.38, 18.27, and 5.22% (*C. longa*) and 27.71, 7.27, 8.08, and 2.71% (*Z. officinale*). The difference in the composition of these two EOs is that oxygenated sesquiterpenes (i.e., α-turmerone (10.28%), germacrone (3.34%), curlone (5.15%), and *ar*-turmerone (9.93%)) were found in *C. longa* EO while monoterpenes including β-phellandrene (14.78%) and camphene (6.94%) were found in *Z. officinale* EO. 8,9-Dehydro-9-formyl cycloisolongifolene (29.31%), curdione (13.52%), and germacrone (8.95%) were shown as major components of the EO extracted from another curcuma species, *C. zedoaria*.

The *C. longa* EO was chosen for further investigations because it was easy to obtain in a high amount as being present in the marc after turmeric starch extraction. The first fractionation using column chromatography with silica gel and gradients of eluents (n-hexane-ethyl acetate) allowed to obtain two important groups, CF1 with sesquiterpenes and CF5 with oxygenated sesquiterpenes. After the second column chromatography using silver nitrate impregnated silica gel of both fractions, three compounds, β-sesquiphellandrene, *ar*-curcumene, and curlone with a purity respectively of 96.9, 97.4, and 91.7%, were purified.

**Figure 2:**
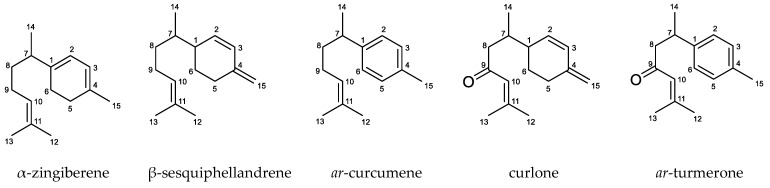
Chemical structures of five major compounds tested from *C. longa* EO.

These isolated compounds along with two commercially available ones, α-zingiberene and *ar*-turmerone (chemical structures in Figure 2), were analyzed for anti-trypanosomal activity and cytotoxicity. The results are summarized in Table 3. Curlone with an IC_50_ of 1.38 µg/mL (6.32 µM) against *Tbb* bloodstream form and SI > 10 compared to cytotoxicity on mammalian cells could explain a part of the observed activity of the EO (IC_50_ = 3.17 nL/mL). This compound may be considered as a promising model for the development of a new treatment of HAT.

## 3. Discussion

This is the first time that these thirty-seven EOs extracted from Vietnamese plants were described for anti-trypanosomal activity in vitro. Some of these EOs were already reported to be tested on the same model, such as *D. ambrosioides*, *M. alternifolia*, and *O. gratissimum* [17,18,19], but they were extracted from plants collected in other countries with possibly different compositions. Based on a preliminary screening, half of them showed a potential activity on *Tbb* with less than 3% of viable parasites at 25 nL/mL (or 10 nL/mL for four of them). Within these nineteen EOs, the one extracted from *C. cassia* revealed the strongest effect (IC_50_ = 1.77 ± 0.15 nL/mL), 17 EOs showed moderate activity with IC_50_ values between 2–20 nL/mL, and one EO extracted from *P. indica* showed less interesting activity with an IC_50_ value of 21.29 ± 1.38 nL/mL. In order to identify the most selective EOs, the cytotoxicity on two different mammalian cell lines, WI38 and J774, was evaluated in parallel. Four samples extracted from *C. longa*, *C. zedoaria*, *L. cubeba*, and *Z. officinale* displayed SI from 14 to > 19 (WI38) and 11 to > 19 (J774). GC analyses led to the identification of more than 85% of their components. We observed the predominance of sesquiterpenes in EOs extracted from rhizomes of the three Zingiberaceae species (*C. longa*, *C. zedoaria*, and *Z. officinale*) while monoterpenes were the major compounds in the EO extracted from fruits of a Lauraceae species (*L. cubeba*).

In the literature, monoterpenes were already shown to be the major compounds of EOs extracted from *L. cubeba*, although the identified components were different. Indeed, EOs extracted from fruits of *L. cubeba* collected in China and Taiwan contained mostly citral (neral and geranial) from 57% to 81% [20,21,22,23,24,25]. The EO extracted from another sample collected in China contained limonene oxide (60%) and limonene (12%) [26]. In our study, the EO extracted from fruits of a Vietnamese sample of *L. cubeba* was dominated by six major compounds, citronellal (43.10%), isopulegol (11.10%), limonene (8.72%), pulegol (6.52%), linalool (5.60%), and citronellol (5.17%). This profile is in agreement with the one extracted from *L. cubeba* collected in India [27]. Four of these six compounds, citronellal, limonene, linalool, and citronellol, are already known for their anti-trypanosomal activity (IC_50_ = 2.76 ± 1.55 [28], 4.24 ± 1.27 [28] or 5.6 ± 1.6 [29], 2.5 [30] and 6.45 ± 4.86 µg/mL [28] respectively on *Tbb* bloodstream form). So, they can explain a part of the observed activity of *L. cubeba* EO (IC_50_ = 2.67 ± 1.12 nL/mL). Nevertheless, the activity of this EO may also partially be due to other compounds or a synergy between these components. It is worth noting that these compounds did not show any cytotoxicity at the highest tested concentration (50 µg/mL for citronellal and citronellol and 100 µg/mL for limonene and linalool) against different mammalian cell lines: CHO, WI38, Balb/3T3 fibroblast, and J774 in those studies [28,29,30].

The *C. zedoaria* EO composition including 8,9-dehydro-9-formyl cycloisolongifolene (29.31%), curdione (13.52%), and germacrone (8.95%) differed from both other EOs but also from previous articles. These publications reported the presence in higher amounts of either monoterpenes such as eucalyptol, *p*-cymene, α-phellandrene, and camphor or other sesquiterpenes such as curzerene, epicurzerene, curdione, curzerenone, and germacrone, depending on the analyzed samples [31]. This variability can be related to the different geographic origins but also to different chemotypes. Germacrone, one of the major compounds identified in our EO, did not show any effect on *Tbb* bloodstream form in the study of Petrelli et al. (IC_50_ > 100 µg/mL) [32], meaning that other components should be responsible for the observed activity of the Vietnamese *C. zedoaria* EO (IC_50_ = 2.51 ± 1.08 nL/mL). Knowing that this EO activity is moderately selective compared to the cytotoxicity on two mammalian cell lines, and that the plant is not difficult to cultivate, *C. zedoaria* EO and its components should be further studied. 

The sesquiterpenes identified in the EO extracted from *Z. officinale* rhizomes were α-zingiberene (27.71%), β-sesquiphellandrene (8.08%), β-bisabolene (7.27%), α-farnesene (3.71%), and *ar*-curcumene (2.71%). Some monoterpenes were also found, such as β-phellandrene (14.78%), camphene (6.94%), and neral (3.16%). This composition is similar to most reports on *Z. officinale* EOs, including samples collected in Brazil, Burkina Faso, Iran, Pakistan, or São Tomé and Príncipe [33,34,35,36,37]. However, rhizomes of samples collected in Australia, India, or Thailand showed a higher quantity of two monoterpenes, citral (neral and geranial) and camphene [38,39,40]. Concerning the anti-trypanosomal activity of these major components, only camphene was tested in the study of Mulyaningsih et al. However its activity was not significant against *Tbb* bloodstream form (IC_50_ value of 80.66 ± 0.87 μg/mL) [41]. 

Identified compounds of Vietnamese *C. longa* EO were in agreement with the reported chemical profile of samples collected from other countries (e.g. Malaysia, China, India, Pakistan, Bhutan, Brazil, Nigeria, Cameroon, and France) [31]. It contains mainly sesquiterpenes: α-zingiberene—25.38%, β-sesquiphellandrene—18.27%, α-turmerone—10.28%, *ar*-turmerone—9.93%, *ar*-curcumene—5.22%, curlone—5.15%, β-bisabolene—3.38%, and germacrone—3.34%. Regarding the anti-trypanosomal activity of *C. longa*, previous reports only focused on curcumin and other curcuminoids with an IC_50_ around 5 μM for curcumin [42], 9.84 ± 0.84 μM for bisdemethoxycurcumin, and 7.19 ± 1.02 μM for demethoxycurcumin [43]. We report here for the first time the interesting effect of the *C. longa* EO against *Tbb* bloodstream form (IC_50_ = 3.17 ± 0.72 nL/mL) with a good selectivity (SI > 10). Thanks to the purification process, β-sesquiphellandrene, *ar*-curcumene, and curlone could be isolated and tested for anti-trypanosomal activity together with two commercially available compounds, α-zingiberene and *ar*-turmerone. Among them, curlone revealed the most interesting effect against *Tbb* bloodstream form with an IC_50_ value of 1.38 ± 0.45 µg/mL (6.32 ± 2.38 µM) and SI of 31.7 and 18.2 compared to cytotoxicity on two mammalian cell lines, WI38 and J774, respectively. This result showed the potential of curlone in the research for new anti-trypanosomal molecules. α-Zingiberene, β-sesquiphellandrene, and *ar*-curcumene showed a moderate effect with IC_50_ values of 6.91 ± 2.60, 9.89 ± 1.18, and 13.38 ± 2.46 µg/mL respectively. However their activity along with curlone can explain a part of the observed activity for the *C. longa* EO.

Concerning the mechanism of action of curlone, which is not commercially available, there are no data in the literature. Three other related sesquiterpenoids, α-zingiberene, β-sesquiphellandrene, and *ar*-turmerone, showed apoptotic effects on different human cancer cells which was associated with the release of mitochondrial cytochrome c and the activation of capase-3 at concentrations of 120 and 160 µg/mL (α-zingiberene), 5.10 µg/mL (β-sesquiphellandrene), and 40, 80, and 120 µg/mL (*ar*-turmerone) [44,45,46]. The structure of curlone shows the presence of both an exocyclic ethylene group as in β-sesquiphellandrene and a ketone group as in *ar*-turmerone. This suggests that these two groups may play an important role in anti-trypanosomal activity of the compound.

As mentioned before, it is very important to emphasize the composition complexity of EOs, making it difficult to identify active compounds [11]. Indeed, in these mixtures, most components were found in low percentages, while two or three accounted for 20%–70% of the whole oil [47]. Major compounds can be studied easier and are often considered as responsible for EO biological activities. However other constituents could of course contribute to the activity through a higher efficacy, additional and/or synergistic activity [48]. For example, eucalyptol, which was characterized as the major component (accounts from 27% to 55%) in four of our tested EOs: *A. aromaticum*, *E. blanda*, *H. coronarium*, and *L. cubeba* (leaves) (data not shown), was not effective on *Tbb* as shown by its high IC_50_ value (83.15 µg/mL) [49]. However these EOs showed a moderate effect in this study with IC_50_ values in the range of 8–16 nL/mL. On the contrary, antagonistic effects may also occur between constituents of these mixtures [48]. An interesting example is terpinolene, which accounts for 55% of our *C. indica* EO (data not shown). This compound was shown to have a very strong activity against *Tbb* bloodstream form (IC_50_ = 0.035 ± 0.05 μg/mL or 0.041 nL/mL) [50], however the IC_50_ value of our *C. indica* EO was 330 times higher (13.22 ± 4.54 nL/mL). The difference in the experiment design could also be another explanation.

## 4. Materials and Methods 

### 4.1. Chemicals and Materials

*ar*-Curcumene, curlone, and β-sesquiphellandrene were isolated from *C. longa* EO; *ar*-turmerone (purity of 97.9 %-GC) was purchased from Sigma-Aldrich (Bornem, Belgium); α-zingiberene (purity of 95 %-TLC) was acquired from Santa Cruz Biotechnology (Heidelberg, Germany).

Column chromatography (CC) was performed using silica gel 60 (70–230 mesh) (Merck KGaA, Darmstadt, Germany). Thin-layer chromatography (TLC) analysis was done on a sheet pre-coated with silica gel (Merck KGaA, Darmstadt, Germany) and impregnated with AgNO_3_ as described by Sliwowski [51]. The GC-MS analyses were carried out on a TRACE GC 2000 series (Thermo-Quest, Rodano, Italy) and the GC-FID analyses were done on a FOCUS GC (Thermo Finnigan, Milan, Italy).

All used organic solvents were HPLC grade (VWR, Leuven, Belgium).

^1^H-NMR and ^13^C-NMR spectra were recorded in CDCl_3_ on a Bruker Avance spectrometer (Wissembourg, France) at 400 and 100 MHz respectively.

In the anti-trypanosomal and cytotoxicity assays, alamar blue was obtained from Thermo Fisher Scientific (Merelbeke, Belgium); tetrazolium salt MTT (3-(4,5-dimethylthiazol-2-yl)-2,5-diphenyltetrazolium bromide), suramin, and camptothecin were obtained from Sigma-Aldrich (Bornem, Belgium). The fluorescence or absorbance was measured on a spectrophotometer (SpectraMax-Molecular Devices, Berkshire, UK).

### 4.2. PLANTS Collection and Essential Oils Extraction

The thirty-seven EOs used in this study were extracted from Vietnamese plants as described previously [12]. All EOs were dissolved in dimethyl sulfoxide (DMSO) to obtain stock solutions at 20 µL/mL and then further diluted in fresh medium for anti-trypanosomal and cytotoxicity assays.

### 4.3. Parasites, Cells, and Media

*Trypanosoma brucei brucei*, although not infecting humans because of its susceptibility to the innate immune system, has been used as a good predictive model in a first screening for the identification of anti-trypanosomal compounds [52,53]. Bloodstream forms of this parasite (*Tbb*, strain 427) were grown in HMI-9 medium (IMDM-Gibco-Thermo Fisher Scientific, Merelbeke, Belgium-supplemented with 10% heat-inactivated fetal bovine serum (Sigma-Aldrich, Bornem, Belgium) and bloodstream form supporting factors) [54].

The human non-cancer fibroblast cell line WI38 (ATCC Number CCL-75 – Standards, UK) and macrophage-like murine cell line J774 (ECACC Number 91051511 – Public Health England, UK) were grown in DMEM and RPMI medium (Gibco-Thermo Fisher Scientific, Merelbeke, Belgium or Sigma-Aldrich, Bornem, Belgium), respectively, supplemented with 10% fetal bovine serum and penicillin-streptomycin (100 UI/mL) (Sigma-Aldrich, Bornem, Belgium).

Parasites and cells cultures were maintained at 37 °C in 5% CO_2_ incubator.

### 4.4. Anti-Trypanosomal Assay

The assay was performed in 96-well plates as previously described [28]. The primary screening was repeated two times in triplicate at the concentrations of 50 nL/mL and 25 nL/mL (for 33 EOs) or 10 nL/mL and 5 nL/mL (for 4 EOs). Fifty µL of parasite culture (5 x 10^4^ parasites/mL) was added with 50 µL of diluted EOs in each well. Ten µL of alamar blue (diluted with PBS at the ratio 1:1) was added to each well after 72 h of incubation and the plates were further incubated for 4 h. The fluorescence of the reduced reagent was measured on a spectrophotometer at 530 nm excitation and 590 nm emission wavelengths. Suramin was used as positive control. The EOs that inhibited more than 50% of the parasite growth at 25 or 10 nL/mL were analyzed for IC_50_ determination. Samples were tested in eight serial three-fold dilutions ranging from 50–0.02 nL/mL, except *Cinnamomum cassia* EO, *Curcuma zedoaria* EO, and *Zingiber zerumbet* EO (ranging from 10–0.005 nL/mL) and *Dysphania ambrosioides* EO (ranging from 5–0.002 nL/mL) in duplicate. IC_50_ values were calculated from dose response growth inhibition curves using Microsoft Excel files and mean IC_50_ values were obtained from at least three repetitions.

### 4.5. Cytotoxicity Assay

The cytotoxicity assays were performed as described previously [12] with concentrations ranging from 50 to 1.40 nL/mL (dilution of 1.67). The selectivity index (SI) values were calculated using the formula:

SI = IC_50_ on mammalian cells/IC_50_ on protozoan parasites

### 4.6. Essential Oils Analysis

Four EOs were analyzed as explained in our previous publication [12].

### 4.7. Components Isolation 

**Figure 3:**
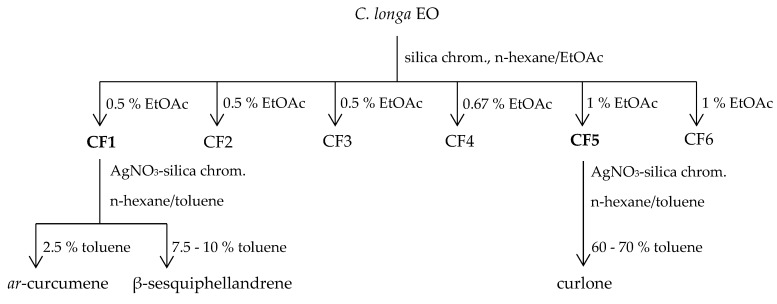
Isolation of β-sesquiphellandrene, *ar*-curcumene, and curlone from *C. longa* EO.

*C. longa* EO obtained by hydro-distillation was subjected to column chromatography on silica gel 60 (70–230 mesh) using n-hexane/ethyl acetate (EtOAc) gradients as the eluent to yield six fractions (CF1-CF6). CF1 and CF5 were further separated to obtain three compounds by column chromatography using AgNO_3_-impregnated silica gel as stationary phase because argentation chromatography is known for the purification of *cis-trans*-isomers or positional isomers mixtures [55,56,57,58]. This separation relies on the weak interactions between silver ions and the *π*-orbital of olefins in which *cis*-olefinic structures complex more tightly with silver ions than the *trans*-isomers [55]. We modified the procedure of Denyer et al. [58] with 10% (*w*/*w*) of silver nitrate instead of 25%, and a gradient of n-hexane and toluene was preferred to hexane and benzene (Figure 3). These isolated compounds were confirmed as β-sesquiphellandrene, *ar*-curcumene, and curlone using NMR in comparison with data from previous reports [59,60,61] and their purity was checked by GC-FID. 

## 5. Conclusions

Our results highlighted for the first time the interesting anti-trypanosomal activity of four EOs extracted from Vietnamese plants, *C. longa*, *C. zedoaria*, *L. cubeba*, and *Z. officinale*. Monoterpenes were major components of *L. cubeba* EOs, while the three other EOs contained mostly sesquiterpenes. Among the five major compounds of the *C. longa* EO, curlone was the most active and selective compound. This compound can explain a part of the observed activity of the EO. Its activity should be further investigated in the research for new anti-trypanosomal compounds.

## Figures and Tables

**Table 1:** Anti-trypanosomal activity, cytotoxicity, and selectivity indices of the 19 selected EOs.

Plant Species (Studied Parts)	Anti-Trypanosomal Activity (IC_50_ nL/mL)	Cytotoxicity (IC_50_ nL/mL)			
		WI38	SI	J774	SI
*Amomum aromaticum* (fruits)	8.75 ± 1.25	47.31 ± 0.30	5.4	22.68 ± 3.22	2.6
*Artemisia annua* (leaves)	8.99 ± 1.18	45.64 ± 1.02	5.1	38.16 ± 0.21	4.2
*Cinnamomum cassia* (stem barks)	1.77 ± 0.15	11.97 ± 0.93	6.8	8.97 ± 0.66	5.1
*Clausena indica* (leaves)	13.22 ± 4.54	>50.00	>3.8	>50.00	>3.8
***Curcuma longa* (rhizomes)**	**3.17 ± 0.72**	**46.00 ± 0.33**	**14.5**	**44.11 ± 3.13**	**13.9**
***Curcuma zedoaria* (rhizomes)**	**2.51 ± 1.08**	**46.64 ± 0.95**	**18.6**	**26.81 ± 1.59**	**10.7**
*Dysphania ambrosioides* (aerial parts)	2.86 ± 0.32	>50.00	>17.5	12.29 ± 2.92	4.3
*Elsholtzia blanda* (leaves)	8.23 ± 1.03	>50.00	>6.1	>50.00	>6.1
*Elsholtzia ciliata* (leaves)	4.26 ± 0.86	48.46 ± 0.12	11.4	13.21 ± 1.48	3.1
*Elsholtzia communis* (leaves)	18.39 ± 3.32	>50.00	>2.7	40.68 ± 3.44	2.2
*Hedychium coronarium* (rhizomes)	9.73 ± 1.43	>50.00	>5.1	30.00 ± 4.06	3.1
*Kaempferia galangal* (rhizomes)	15.78 ± 3.29	>50.00	>3.2	>50.00	>3.2
***Litsea cubeba* (fruits)**	**2.67 ± 1.12**	**>50.00**	**>18.7**	**>50.00**	**>18.7**
*Litsea cubeba* (leaves)	16.47 ± 1.24	>50.00	>3.0	>50.00	>3.0
*Pluchea indica* (leaves)	21.29 ± 1.38	27.47 ± 1.49	1.3	25.05 ± 5.56	1.2
*Pogostemon cablin* (leaves)	4.07 ± 0.98	27.17 ± 3.62	6.7	28.40 ± 1.81	7.0
*Vitex trifolia* (leaves)	3.24 ± 0.79	31.12 ± 2.83	9.6	26.64 ± 0.76	8.2
***Zingiber officinale* (rhizomes)**	**3.10 ± 0.08**	**>50.00**	**>16.1**	**37.52 ± 0.05**	**12.1**
*Zingiber zerumbet* (rhizomes)	6.23 ± 0.73	3.65 ± 0.34	0.6	2.78 ± 0.57	0.5
Suramin	21.53 ± 2.62^a^				
Camptothecin		34.99 ± 9.63 ^a^		7.32 ± 1.29 ^a^	

IC_50_: Mean ± SD calculated in at least triplicate for anti-trypanosomal activity and duplicate for cytotoxicity; ^a^ concentration in ng/mL.

**Table 2:** Chemical composition of the four selected EOs.

No.	Compounds	RI	Relative Percentage (%)				Identification
			*L. cubeba*	*C. zedoaria*	*Z. officinale*	*C. longa*	
1	α-Pinene ^m^	536	0.74	0.11	2.29	-	MS, Co-GC, Ref.
2	α-Thujene ^m^	540	0.18	-	-	-	MS, Ref.
3	**Camphene ^m^**	577	-	0.26	**6.94**	-	MS, Ref.
4	β-Pinene ^m^	621	0.86	0.77	0.16	0.09	MS, Co-GC, Ref.
5	Sabinene ^m^	635	0.83	-	0.19	-	MS, Co-GC, Ref.
6	3-Carene ^m^	665	0.36	-	-	-	MS
7	α-Phellandrene ^m^	679	-	-	0.70	0.08	MS, Co-
8	Myrcene ^m^	681	1.25	-	1.10	t	MS, Co-GC, Ref.
9	α-Terpinene ^m^	697	0.51	-	-	t	MS, Co-GC, Ref.
10	**Limonene ^m^**	714	**8.72**	0.18	2.06	0.19	MS, Co-GC, Ref.
11	**β-Phellandrene ^m^**	727	0.16	-	**14.78**	-	MS
12	Eucalyptol ^m^	727	1.37	1.61	1.79	3.15	MS, Co-GC, Ref.
13	γ-Terpinene ^m^	761	0.52	-	t	t	MS, Co-GC, Ref.
14	*p*-Cymeme ^m^	783	0.10	t	t	t	MS, Co-GC, Ref.
15	Terpinolene ^m^	798	0.31	-	0.26	1.70	MS, Co-GC, Ref.
16	2-Heptanol	844	-	0.14	0.15	t	MS
17	5-Hepten-2-one, 6-methyl-	854	0.35	-	t	-	MS, Co-GC
18	5-Heptenal, 2,6-dimethyl	867	0.68	-	-	-	MS
19	2-Nonanone	903	-	0.43	0.15	t	MS
20	(*E*)-2-Octenal	940	-	-	t	-	MS
21	2-Octanol	941	-	t	-	-	MS
22	*p*-Cymenene ^m^	946	-	-	-	t	MS
23	1-Octen-3-ol	969	-	t	-	-	MS, Ref.
24	δ-Elemene ^s^	980	-	0.30	t	t	MS
25	Cyclosativene ^s^	986	-	-	t	-	MS
26	**Citronellal ^m^**	993	**43.10**	-	0.30	0.14	MS, Co-GC, Ref.
27	α-Copaene ^s^	999	-	-	0.31	-	MS
28	Decanone	1006	-	t	-	-	MS
29	Camphor ^m^	1020	-	4.18	t	-	MS, Co-GC, Ref.
30	2-Nonanol	1038	-	2.16	0.19	0.21	MS
31	**Linalool ^m^**	1063	**5.60**	0.22	0.48	t	MS, Co-GC, Ref.
32	*cis*-α-Bergamotene ^s^	1065	-	-	t	0.27	MS
33	**Pulegol ^m^**	1072	**6.52**	-	-	-	MS
34	**Isopulegol ^m^**	1082	**11.10**	-	-	-	MS, Ref.
35	*trans*-α-Bergamotene ^s^	1091	-	-	-	0.12	MS, Ref.
36	β-Elemene ^s^	1096	-	4.85	0.34	0.22	MS
37	β-Caryophyllene ^s^	1100	-	3.79	0.43	t	MS, Co-GC, Ref.
38	2-Undecanone	1106	-	-	0.39	t	MS
39	Terpinene-4-ol ^m^	1109	2.58	0.31	0.22	0.13	MS, Co-GC, Ref.
40	γ-Elemene ^s^	1142	-	0.32	-	0.09	MS
41	α-Himachalene ^s^	1153	-	-	-	t	MS
42	γ-Gurjunene ^s^	1160	-	-	-	t	MS
43	α-Humulene ^s^	1168	-	1.28	-	t	MS, Co-GC, Ref.
44	(*E*)-β-Farnesene ^s^	1174	-	-	0.26	0.61	MS
45	Neral ^m^	1186	-	-	3.16	-	MS, Co-GC
46	α-Terpineol ^m^	1203	0.62	0.23	1.78	0.48	MS, Co-GC, Ref.
47	Borneol ^m^	1208	-	-	1.35	-	MS, Co-GC, Ref.
48	Germacrene D ^s^	1206	-	1.99	-	t	MS, Ref.
49	α-Muurolene ^s^	1217	-	-	1.44	-	MS
50	β-Selinene ^s^	1218	-	1.76	-	-	MS
51	β-Chamigrene ^s^	1223	-	1.47	-	-	MS
52	**α-Zingiberene ^s^**	1236	-	-	**27.71**	**25.38**	MS, Co-GC
53	**β-Bisabolene ^s^**	1238	-	-	**7.27**	**3.38**	MS, Ref.
54	α-Cubebene ^s^	1248	-	-	0.23	-	MS
55	(*E,E*)-α-Farnesene ^s^	1259	-	-	3.71	0.36	MS
56	**Citronellol ^m^**	1274	**5.17**	-	-	-	MS, Co-GC, Ref.
57	**β-Sesquiphellandrene ^s^**	1279	-	-	**8.08**	**18.27**	MS
58	***ar*-Curcumene ^s^**	1280	-	-	**2.71**	**5.22**	MS
59	ζ-Elemene ^s^	1323	-	2.47	0.55	-	MS
60	Geraniol ^m^	1351	-	-	0.45	0.25	MS, Co-GC, Ref.
61	Curzerene ^s^	1366	-	4.87	-	-	MS
62	Epiglobulol ^s^	1468	-	0.57	-	-	MS
63	(*E*)-Nerolidol ^s^	1536	-	-	0.32	-	MS, Co-GC, Ref.
64	Elemol ^s^	1569	-	-	0.18	-	MS
65	Ledene oxide ^s^	1583	-	0.25	-	-	MS
66	Bisabolone ^s^	1595	-	-	-	1.08	MS
67	Spathulenol ^s^	1607	0.21	-	-	-	MS
68	ar-Turmerol ^s^	1658	-	-	-	1.60	MS
69	α-Turmerone ^s^	1667	-	-	-	10.28	MS
70	Bisabolol ^s^	1670	-	-	-	0.87	MS
71	**8,9-Dehydro-9-formyl cycloisolongifolene ^s^**	1703	-	**29.31**	-	-	MS
72	**Germacrone ^s^**	1710	-	**8.95**	-	**3.34**	MS
73	**Curlone ^s^**	1723	-	-	-	**5.15**	MS
74	***ar*-Turmerone ^s^**	1739	-	-	-	**9.93**	MS, Co-GC
75	**Curdione ^s^**	1792	-	**13.52**	-	-	MS
76	Farnesol ^s^	1838	-	-	0.20	-	MS, Ref.
	**Total identified**		**91.84**	**86.30**	**92.63**	**92.59**	

^m^: monoterpene; ^s^: sesquiterpene; t: trace (peak area less than 0.05%); RI: the retention index was calculated using a homologous series of fatty acid methyl esters C5–C27; MS: mass spectra (matching coefficient > 700 compared with NIST database); Co-GC: co-injection with pure compound; Ref.: reference [15,16]; major compounds are in bold.

**Table 3:** Anti-trypanosomal activity and cytotoxicity of five pure compounds identified in *C. longa* EO.

Compounds	Anti-Trypanosomal Activity (IC_50_ µg/mL)	Cytotoxicity (IC_50_ µg/mL)			
		WI38	SI	J774	SI
α-Zingiberene	6.91 ± 2.60	28.50 ± 1.43	4.1	29.64 ± 2.54	4.3
β-Sesquiphellandrene	9.89 ± 1.18	19.11 ± 1.58	1.9	21.02 ± 2.72	2.1
*ar*-Curcumene	13.38 ± 2.46	23.15 ± 1.36	1.7	24.03 ± 2.64	1.8
**Curlone**	**1.38 ± 0.52**	**43.64 ± 2.45**	**31.7**	**25.06 ± 3.47**	**18.2**
*ar*-Turmerone	28.83 ± 3.93	43.39 ± 3.89	1.5	44.62 ± 1.41	1.6
Suramin	21.53 ± 2.62 ^a^				
Camptothecin		34.99 ± 9.63 ^a^		7.32 ± 1.29 ^a^	

IC_50_: Mean ± SD calculated in at least triplicate for anti-trypanosomal activity and duplicate for cytotoxicity; ^a^ concentration in ng/mL.
